# MAT as a promising therapeutic strategy against triple-negative breast cancer via inhibiting PI3K/AKT pathway

**DOI:** 10.1038/s41598-023-39655-9

**Published:** 2023-07-31

**Authors:** Shijie Wei, Yubao Zhang, Xiaoran Ma, Yan Yao, Qinqin Zhou, Wenfeng Zhang, Chao Zhou, Jing Zhuang

**Affiliations:** 1 Institute of Integrated Medicine, Qingdao University, Qingdao, 266071 China; 2 Department of Oncology, The Affiliated Qingdao Hiser hospital of Qingdao University (Qingdao Hospital of Traditional Chinese Medicine), Qingdao, 266071 China; 3 First School of Clinical Medicine, Shandong University of Traditional Chinese Medicine, Jinan, 250355 China; 4 Qingdao Academy of Chinese Medicinal Sciences, Shandong University of Traditional Chinese Medicine, Qingdao, 266114 China; 5 Macau University of Science and Technology, Avenida Wai Long, Taipa, 999078 Macau China; 6 Department of Oncology, Weifang Traditional Chinese Hospital, Weifang, 261000 China

**Keywords:** Breast cancer, Gynaecological cancer

## Abstract

Triple-negative breast cancer (TNBC), a highly aggressive and heterogeneous subtype of breast cancer, lacks effective treatment options. *Sophora flavescens* Aiton, a Chinese medicinal plant, is often used in traditional Chinese medicine to treat cancer. Matrine (MAT) is an alkaloid extracted from *Sophora flavescens*. It has good anticancer effects, and thus can be explored as a new therapeutic agent in TNBC research. We performed bioinformatics analysis to analyze the differentially expressed genes between normal breast tissues and TNBC tissues, and comprehensive network pharmacology analyses. The activity and invasion ability of TNBC cells treated with MAT were analyzed. Apoptosis and cell cycle progression were determined using cytometry. We used Monodansylcadaverine (MDC) staining to determine the condition of autophagosomes. Finally, the expression levels of the key target proteins of the PI3K/AKT pathway were determined using western blotting. The proliferation and invasion ability of MDA-MB-231 and MDA-MB-468 can be effectively inhibited by MAT. The results of flow cytometry indicated that MAT arrested the TNBC cell cycle and induced apoptosis. In addition, we confirmed that MAT inhibited the expression of BCL-2 while up-regulating the expression of cleaved caspase-3. Moreover, enhanced intensity of MDC staining and high LC3-II expression were observed, which confirmed that MAT induced autophagy in TNBC cells. Western blotting showed that MAT inhibited the PI3K/AKT pathway and downregulated the expressions of PI3K, AKT, p-AKT, and PGK1. This study provides feasible methods, which include bioinformatics analysis and in vitro experiments, for the identification of compounds with anti-TNBC properties. MAT inhibited the PI3K/AKT signaling pathway, arrested cell cycle, as well as promoted cell apoptosis and autophagy. These experiments provide evidence for the anti-TNBC effect of MAT and identified potential targets against TNBC.

## Introduction

According to the 2020 statistics, there are 2.26 million new cases of breast cancer, 30% of newly diagnosed cases in women and accounting for 15% of cancer-related deaths worldwide^1^. Triple-negative breast cancer (TNBC) is associated with a very high risk of metastasis, and patients with metastatic TNBC have a median overall survival of only 18 months^2^. Progesterone receptor, human epidermal growth factor receptor 2 and estrogen receptor are not expressed in TNBC, and hence, patients benefit little from these treatments. With increasing understanding of TNBC-related mechanisms, the survival rate of patients has improved by means of surgery, radiotherapy, chemotherapy, and neoadjuvant therapy. However, the adverse reactions and increasing drug resistance associated with these treatment strategies still pose a huge threat to the prognosis of patients. These traditional methods have been unable to meet the clinical requirements of treatment, therefore, research focused on identifying more effective treatment methods to decelerate the progression of TNBC has become a priority.

Natural drugs have the characteristics of multi-target and multi-path intervention^3^. At present, some natural drug extracts, such as paclitaxel^4^ and curcumin^5^ with extensive pharmacological effects and low side effects have shown good efficacy in TNBC. *Sophora flavescens* Aiton is a traditional Chinese medicine herb derived from the dried root of *Sophora flavescens* Aiton, a medicinal plant of the *Leguminosae* family and the plant name has been checked with “The Plant List” (www.theplantlist.org). *Sophora flavescens* Aiton can alleviate Parkinson's disease^6^ and treatment for solid tumors, inflammation, and other diseases^7^. Matrine (MAT) is a compound extracted from *Sophora flavescens* Aiton, which has antitumor, anti-inflammatory, anti-viral, neuroprotective, and obesity-inhibiting potential^8–11^. MAT can suppress gastric cancer, acute leukemia, bladder cancer, and other cancers by inhibiting the PI3K/AKT pathway^12–14^. Relevant studies have confirmed that MAT can exert anti-tumor functions with inducing autophagy^15,16^. High expression of autophagy-related 7 (Atg7) has a better prognostic effect, as Atg7 expression is considerably lower in TNBC than in normal breast patients^17^. Moreover, the inhibition of autophagy can help fight tumors and reverse drug resistance in tumor cells^18^. These reports suggest that autophagy plays an important role in antitumor activity. MAT can promote apoptosis and cycle arrest of tumor cells by inducing autophagy^19^. However, it has not yet been reported if MAT can induce autophagy in TNBC, and its anti-TNBC mechanism has not been explored. Therefore, it is of great significance to assess the effect of MAT on cell proliferation and autophagy in TNBC.

Recent advances in biomedical technology have facilitated a deeper understanding of cancer. Through the rapid development of multi-high-throughput gene chip sequencing technology, we have a better understanding of genetic changes that are associated with cancer. This improves the accuracy in the early diagnosis of tumors by traditional tissue pathology. Differentially expressed genes (DEGs) between normal breast and breast cancer tissues can also be identified using bioinformatics. Gene Expression Omnibus (GEO), a high-throughput database, provides accumulated data, and the analysis of differences between gene chips in the database can help us gain a deeper understanding of disease markers^20^. Molecular docking technology facilitates the understanding of the interactions between molecules and can provide a theoretical basis for the prediction of drug intervention targets.

This study used bioinformatics analysis and comprehensive network pharmacology analyses, and explore the molecular mechanism of MAT intervention in vitro experiments in TNBC. The results of this study may provide new strategies and evidence for TNBC treatment.

## Materials and methods

### Reagent

MAT (purity: ≥ 98%) was purchased from Shanghai Yuanye Bio-Technology Co., LTD, (China) and dissolved in phosphate buffered saline (PBS). MDA-MB-231 and MDA-MB-468 cell lines were obtained from American Type Culture Collection (ATCC, United States). PI3K (67071-1-Ig), P-AKT (66444-1-Ig), AKT (60203-2-Ig), and GAPDH (60004-1-Ig) were purchased from Proteintech (Wuhan, China) PGK1 (sc-130335) and LC3- II (sc-271625) were purchased from Santa Cruz Biotechnology, Inc., (Texas, United States), and BCL-2 (ab182858) and cleaved caspase-3 (ab32042) were purchased from Abcam (Cambridge, UK).

### Cell culture

TNBC cells (MDA-MB-231 and MDA-MB-468) were cultured in a cell incubator at 5% CO_2_, 37 °C, and 95% humidity, and the cells in the logarithmic growth phase were used for the experiments.

### Determination of cell viability

TNBC cells were cultured in 96-well plates with a seeding density of 3 × 10^3^ cells/well, they were incubated with 0, 2, 4, 8 and 12 mM MAT for 24, 48 and 72 h, respectively, after the cells adhered. The conditioned medium was replaced with which containing 20 μL of 3-(4,5-dimethylthiazol-2-yl)-2,5-diphenyltetrazolium bromide (MTT) solution. The MTT solution was discarded after further incubation for 4 h, followed by the addition of DMSO solution (150 μL) to each well. After incubation in the dark for a quarter of an hour, the data measured by absorbance at 490 nm were obtained in a microplate reader (BioTek, USA).

### Cell migration

When the TNBC cells in the 6-well plates reached 80–90% confluence, a pipette tip was used to scratch each well, perpendicular to the bottom of the culture plate, the cells were washed thrice with PBS. After treating the cells with MAT (0, 2 and 4 mM) and culturing in DMEM without fetal bovine serum for 24 h, cell migration was recorded using a light microscope (CKX53, Olympus Corporation, Japan) in the same field of view. Images were processed using the ImageJ (National Institutes of Health, MD, USA).

### Cell invasion

We diluted Matrigel (BD Biosciences, NJ, USA) by medium without fetal bovine serum, in a ratio of 1:6, added 100 μL of the mixed solution to the upper chamber, and placed the chamber in a cell incubator for solidification. The basement membrane was then hydrated by medium without fetal bovine serum for half an hour. The concentration of the cell suspension was adjusted to 1 × 10^5^/mL, and 200 μL of the suspension containing MAT (0, 2 and 4 mM) was added to the upper chamber. Then the upper chamber was immersed in a plate added with 500 µL of complete medium (10% fetal bovine serum). Residual medium was discarded along with the cells in the upper chamber. The chambers were soaked in paraformaldehyde for 40 min, and then soaked in 0.1% crystal violet solution for a quarter. The stained chambers were washed with PBS, then observed and photographed with a microscope.

### Determination of apoptosis and cell cycle by flow cytometry

After treatment of TNBC cells with MAT (0, 2 and 4 mM) for 24 h, the Annexin V-FITC kit (BD, 556547) was used to label apoptotic cells, and cell cycle was detected by cell cycle staining kit (YEASEN, 40301ES60). The distribution of cells and apoptosis were detected by flow cytometry (CytoFLEX, USA). Flow data were analyzed using the CytExpert software (Beckman, USA) and FlowJ software (BD).

### Data sources

We retrieved the data associated with "triple-negative breast cancer" from the GEO (www.ncbi.nlm.nih.gov/geo/), and make sure the samples are recorded on GPL6947 and GPL570 platforms. When screening the dataset, our criteria are: the data in the dataset was published or last updated in the past 5 years (i.e., after 2019); in the data set, samples of triple negative breast cancer and healthy breast tissue are included at the same time; the data should come from authoritative research institutions, and the selected dataset for this study is from the Institut Curie and Institute of Oncology Research (IOR, Switzerland). GEO2R is a program provided by the GEO database for the analysis of differences in expression spectrum chips. We grouped the samples based on type and analyzed each chip, followed by saving and screening of the data, with the screening criteria set to p < 0.05, while satisfying | Log_2_ Fold Change |> 1 (Log_2_FC).

### Date analysis

We conduct gene ontology (GO) analysis (p < 0.05) and Kyoto Encyclopedia of Genes and Genomes (KEGG) analysis^21–23^ (p < 0.05) of differential genes through the DAVID (https://david.ncifcrf.gov/) database, and R's ggplot packet to visualize the results. DAVID is a public online bioinformatic repository. It combines bioinformatics information with online tools to effectively explain the gene list in the network environment. DAVID database converts the signal pathway and genetic functions of different genes into visual charts, allowing us to understand these genes more intuitively and comprehensively, which was of great significance to our subsequent analysis and experiments^24^. The Gene Expression Profiling Interactive Analysis (GEPIA) databases are online visualization tools for The Cancer Genome Atlas database (TCGA)^25^. We used the online analysis software GEPIA (http://gepia.cancer-pku.cn) for prognostic analysis and selected the genes with p value < 0.01 for the subsequent analysis and protein–protein interaction analysis was carried out by String(https://cn.string-db.org/) and Cytoscape.

### Molecular docking

The molecular structure of MAT and NG52 ware derived from PubChem (http://www.pubchem.ncbi.nlm.nih.gov) and saved as SDF (CID: 91466 and 2856).The crystal structures of the targeted protein war derived from the RCSB (https://www.rcsb.org/) and saved in PDB format (The IDs of PGK1 and PI3KCA are respectively 4O33^26^ and 2RD0^27^). We pretreated the receptors before docking as follows: remove water of crystallization and non-standard peptide chains, combine non-polar hydrogen, combine lone pair electrons, remove solvent molecules, and add all hydrogen atoms. After the pretreatment of ligands and receptors, Autodock vina 1.2.3 is used for molecular docking^28–30^. The docking experiment between MAT and PGK1, NG52 and PGK1 was conducted by selecting a 20 × 20 × 20 docking region centered on the key amino acid residue. MAT and PIK3CA used blind docking methods to search the entire protein space for the optimal binding site. The algorithm exhaustiveness is 16. Adopting semi flexible docking: the ligand is completely flexible, and the protein is rigid.

### Monodansylcadaverine (MDC) staining of autophagosomes

TNBC cells which were cultured to 65% confluence in 6-well plates and treated with MAT for 24 h and autophagy was detected using a detection kit (Beyotime, C3019S). The MDC staining solution was added according to the prescribed amount, followed by incubation at 37 °C for 25 min with wrapping in tin foil. Take out the stained cells and wash with PBS for 3 times in a dark environment. Using a fluorescence microscope, the resulting green fluorescence was observed under ultraviolet light, and images were captured.

### Western blot

TNBC cells were cultured to 70% confluence in 6-well plates, followed by treatment with MAT (0, 2 and 4 mM) for one day. After washed with pre-cold PBS, the cells were cleaved with RIPA cell lysate buffer containing 1% protease inhibitor and 1% phosphatase inhibitor, followed by the extraction of total protein. The collected total protein was centrifuged at 12,000 rpm for a quarter. After that, the protein concentration was obtained by the BCA kit. Loading buffer was added to the samples, and the mixture was allowed to boil to denature the proteins, after which western blotting was performed.

All antibodies were diluted according to the recommended antibody concentration, and the diluted antibodies were stored at 4 °C. SDS-PAGE was used to separate the same amount (40 μg) of protein, and the separated target protein was transferred to PVDF film on ice. Next, degreasing milk powder (5%) was added to unphosphorylated protein, and Bovine Serum Albumin (5%) was added to phosphorylated protein, at room temperature for 1.5 h, respectively. Protein was incubated at 4 °C for 12 h with primary antibody, then it was incubated for 1 h with secondary antibody. Finally, the results were detected using the ECL kit.

### Statistical analysis

GraphPad Prism 9 was used to analyze the data. The student’s t-test or single-factor square difference analysis (ANOVA) was selected for statistical analysis of the obtained data, and statistical results are reported as mean ± standard deviation ($\overline{x }$ ± s). For all statistical analyses, *p < 0.05, **p < 0.01, ***p < 0.001.

## Results

### MAT inhibits TNBC cell viability

**Figure 1:**
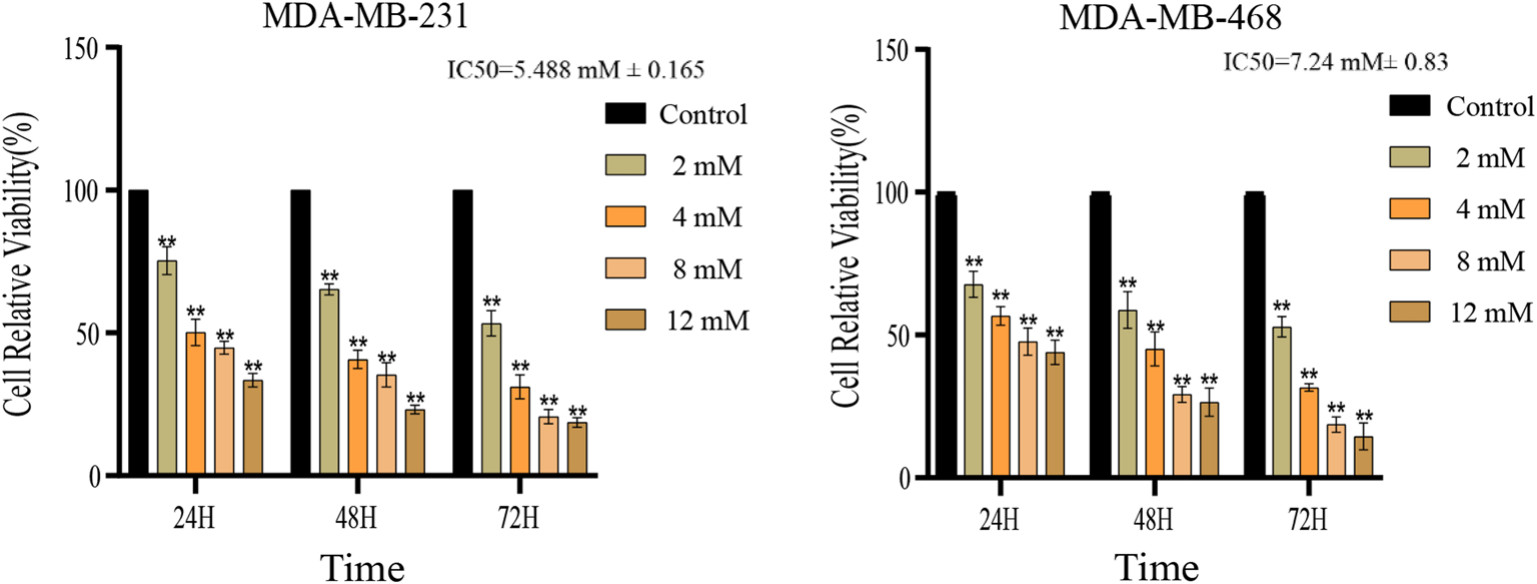
Effects of matrine (MAT) on TNBC cell proliferation. Viability was determined by MTT assay of TNBC cells. The IC50 of MDA-MB-231 cells is 5.488 mM ± 0.165, while that of MDA-MB-468 cells is 7.24 mM ± 0.83.

After treatment with MAT for 24 h, the TNBC cells morphology changed significantly, TNBC cells began to shrink and deform, and vacuoles appeared in the cells. MTT results showed that MAT inhibited the proliferation of TNBC cells and its inhibitory effect was concentration- and time-dependent (Fig. 1). In order to avoid interference of MAT cytotoxicity on TNBC cells, we reduced the intervention concentration in subsequent experiments.

### MAT inhibits the migration ability and invasion ability

**Figure 2:**
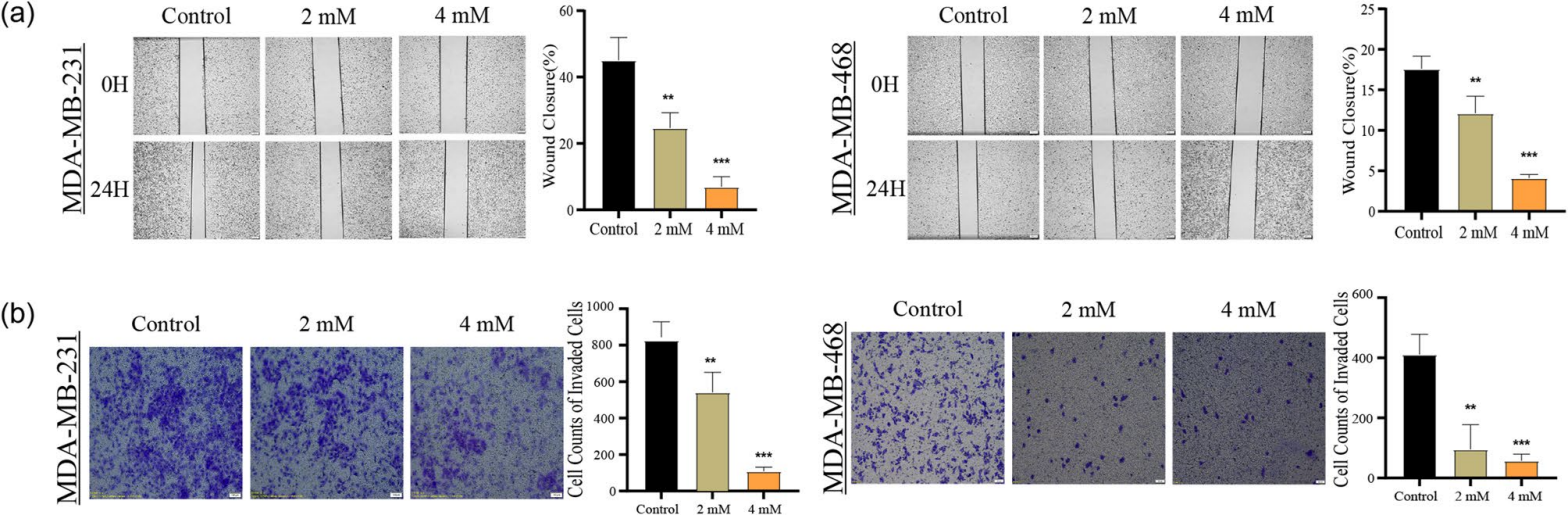
Matrine (MAT) inhibits the migration and invasion of TNBC cells. (**a**) The degree of healing of the scratched part of TNBC cells after treatment with MAT (0, 2 and 4 mM) for 24 h intervention. (**b**) Transwell assay was used to detect the invasive ability of cells.

As the MAT concentration increased, the degree of healing of the scratched part decreased (Fig. 2a). Moreover, we found that the TNBC cells treated with high concentrations of MAT had significantly fewer cells capable of invading Matrigel than the control group (Fig. 2b). This indicated that MAT effectively inhibited the migration and invasion abilities of MDA-MB-231 and MDA-MB-468.

### MAT induces apoptosis and interferes with the cell cycle

**Figure 3:**
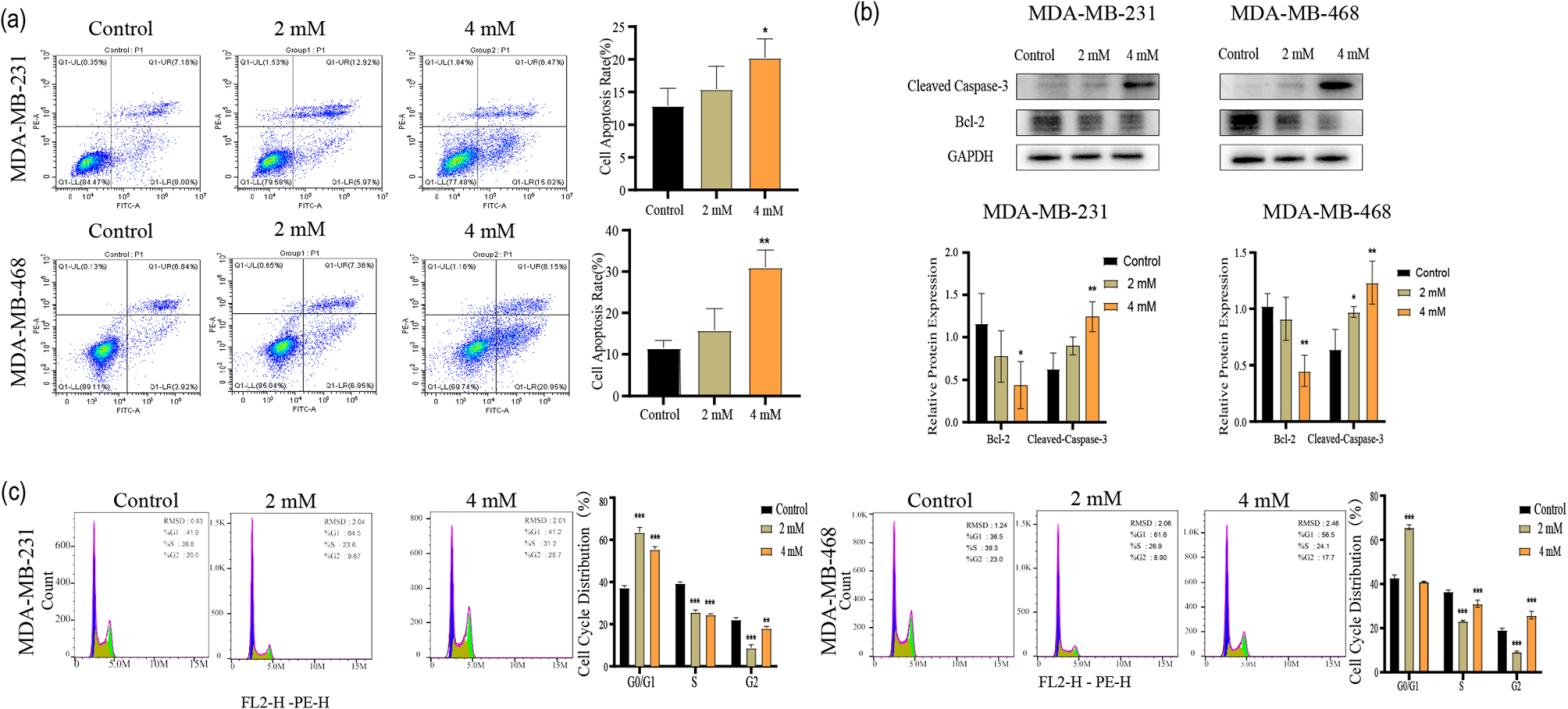
Matrine (MAT) induces apoptosis and arrests cell cycle in TNBC cells. (**a**) Flow cytometry was used to detect changes in the apoptosis of TNBC cells after treatment with MAT (0, 2 and 4 mM) for 24 h. (**b**) After 24 h of treatment with MAT (0, 2 and 4 mM), the cells were analyzed by Western blot to determine the expression level of apoptosis-related proteins. The original imaging representing immunoblots ware shown in Supplementary Fig. S1. (**c**) Cell cycle changes after treated with MAT (0, 2 and 4 mM) for 24 h. *p < 0.05, **p < 0.01, ***p < 0.001, compared with the control group.

In this study, compared with control group, TNBC cells treated with high concentrations of MAT proliferate more slowly, therefore, MAT may have an impact on TNBC cell cycle. We used flow cytometry to observe apoptosis and cell cycle of MAT-treated TNBC cells. The percentage of apoptotic MDA-MB-231 (12.93% ± 2.635%, 15.44% ± 3.491%, 20.25% ± 2.869%) and MDA-MB-468 (11.59% ± 1.777%, 15.81% ± 5.210%, 31.02% ± 4.218%) cells increased significantly with an increase in MAT concentration. Meanwhile, MAT inhibited the expression of BCL-2 while up-regulating the expression of cleaved caspase-3 in concentration-dependent manners (Fig. 3a,b). Compared with that in the control group, the ratio of MDA-MB-231 (37.27% ± 1.002%, 63.53% ± 2.386%, 55.50% ± 1.249%) and MDA-MB-468 (42.87% ± 1.266%, 65.73% ± 1.097%, 41.07% ± 0.2309%) cells in the G0/G1 phase in the MAT group (2 and 4 mM) increased significantly, while the ratio of S phase to G2 phase decreased (Fig. 3c). These findings verified our hypothesis that MAT can induce apoptosis and arrest the cell cycle in TNBC cells.

### Data analysis of DEGs

**Figure 4:**
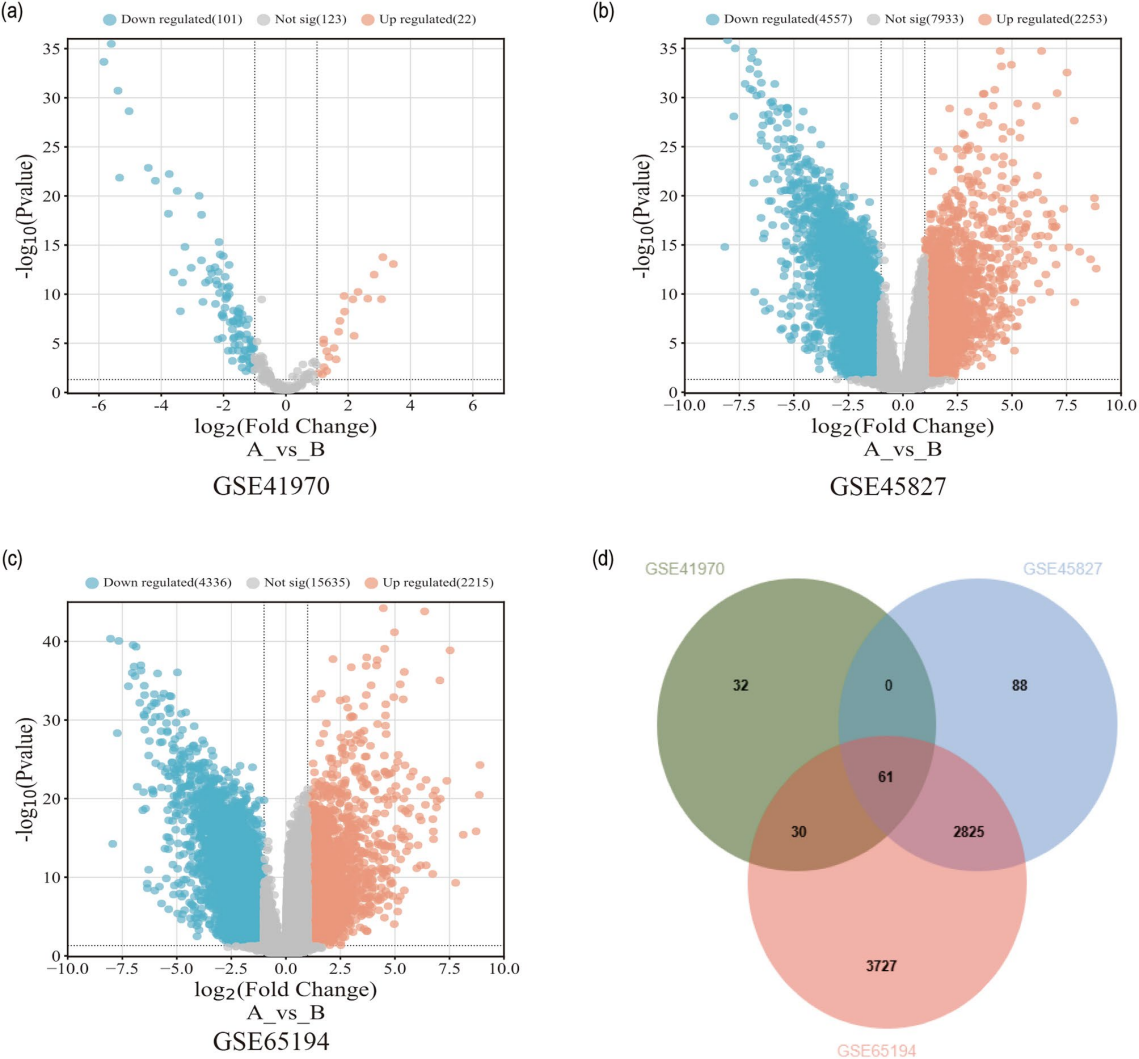
Identification of DEGs in breast cancer. DEGs in TNBC tumor tissues and normal breast tissues from GSE41970 (**a**), GSE45827 (**b**), and GSE65194 (**c**) were showed by Volcano plots. (**d**) The common DEGs in three data sets were presented by a Venn diagram.

We further elucidate the related mechanisms and important targets of MAT intervention in TNBC cells through bioinformatics. In the GEO database, three microarray datasets (GSE41970, GSE45827, and GSE65194) were selected (Fig. 4a–c), including 81 normal breast samples and 247 TNBC samples. The microarray datasets were analyzed by GEO2R, and the analyzed data were preserved and screened. The screening criteria were p < 0.05 and | Log_2_FC |> 1. After the screening was completed, we analyzed the results using a Venn diagram, and 61 DEGs were obtained (Fig. 4d).

**Figure 5:**
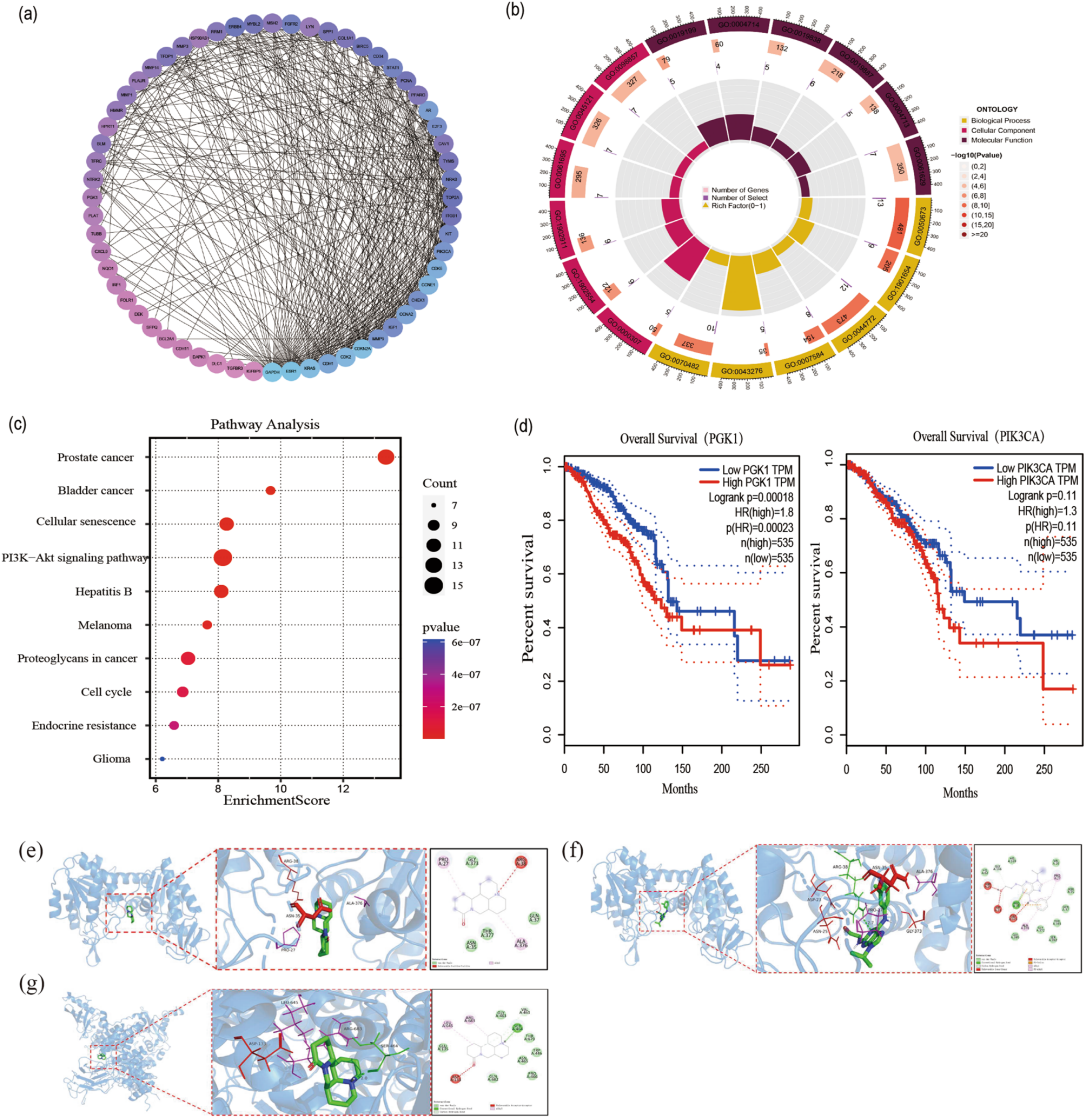
Visualization of comprehensive pharmacological results of differentially expressed genes. (**a**) Protein–protein interaction of potential target proteins. (**b**) GO analysis of differentially expressed genes (p < 0.05). (**c**) The top 10 KEGG pathways for potential targets (p < 0.05). (**d**) Prognostic value analysis of PGK1 and PIK3CA in GEPIA. (**e**) MAT docked with molecules of PGK1. (**f**) NG52 docked with molecules of PGK1. (**g**) MAT docked with molecules of PIK3CA. The blue cartoon model represents the receptor, the green bat model represents the ligand, and the red bat model represents the key residue binding to ASN35.

To further understand the DEGs between TNBC and normal breast tissue. A protein–protein interaction network with 60 nodes and 854 edges was constructed (Fig. 5a). At the same time, DAVID database was used for GO analysis of the 61 DEGs to explore the molecular functions of these genes. The cut-off criterion is p < 0.05. We found that these DEGs were mainly enriched in cell proliferation, cell cycle, and PI3K/AKT signal transmission function (Fig. 5b). Through the analysis of the KEGG pathway enrichment of the DEGs (p < 0.05), the key genes of MAT regulation were found to be involved in the PI3K/AKT pathway (Fig. 5c). The univariate cox analysis (p < 0.01) and prognostic value analyses showed that the *PGK1*and *PIK3CA*, whose expression ware significantly up-regulated in breast cancer, has a certain prognostic value for breast cancer patients (Fig. 5d).

### MAT molecular docking

Molecular docking is important in drug design and screening. It is usually used to predict protein–ligand interaction. The purpose of our construction of the molecular docking models was to explore the target interaction between MAT and PGK1, NG52 and PGK1, MAT and PIK3CA.

In the process of molecular docking, the macrocystis ligands bind to pocket-shaped regions inside the protein, and the two are well-matched for specific binding effects. The ligand and protein form two alkyl interactions, one adverse binding, and multiple van der Waals interactions. Generally, the lower the affinity, the more stable the binding conformation. Although there is no hydrogen bond link between PGK1 and MAT, its binding affinity is − 6.120 kcal/mol, indicating that the binding of the ligand receptor is stable and the molecular docking results showed that MAT and PGK1 were linked at the ASN-35 site (Fig. 5e). NG52 could reverse the Warburg effect by inhibiting PGK1 kinase activity^31,32^. The binding affinity between NG52 and PGK1 is − 7.565 kcal/mol (Fig. 5f).

MAT binds to the pocket shaped area on the surface of PIK3CA, forming 1 hydrogen bond and 2 hydrophobic bonds Water interaction, adverse hydrogen bond receptor conflict, and multiple van der Waals interactions. The binding affinity of the two reached − 7.469 kcal/mol (Fig. 5g).

### MAT induces autophagy

**Figure 6:**
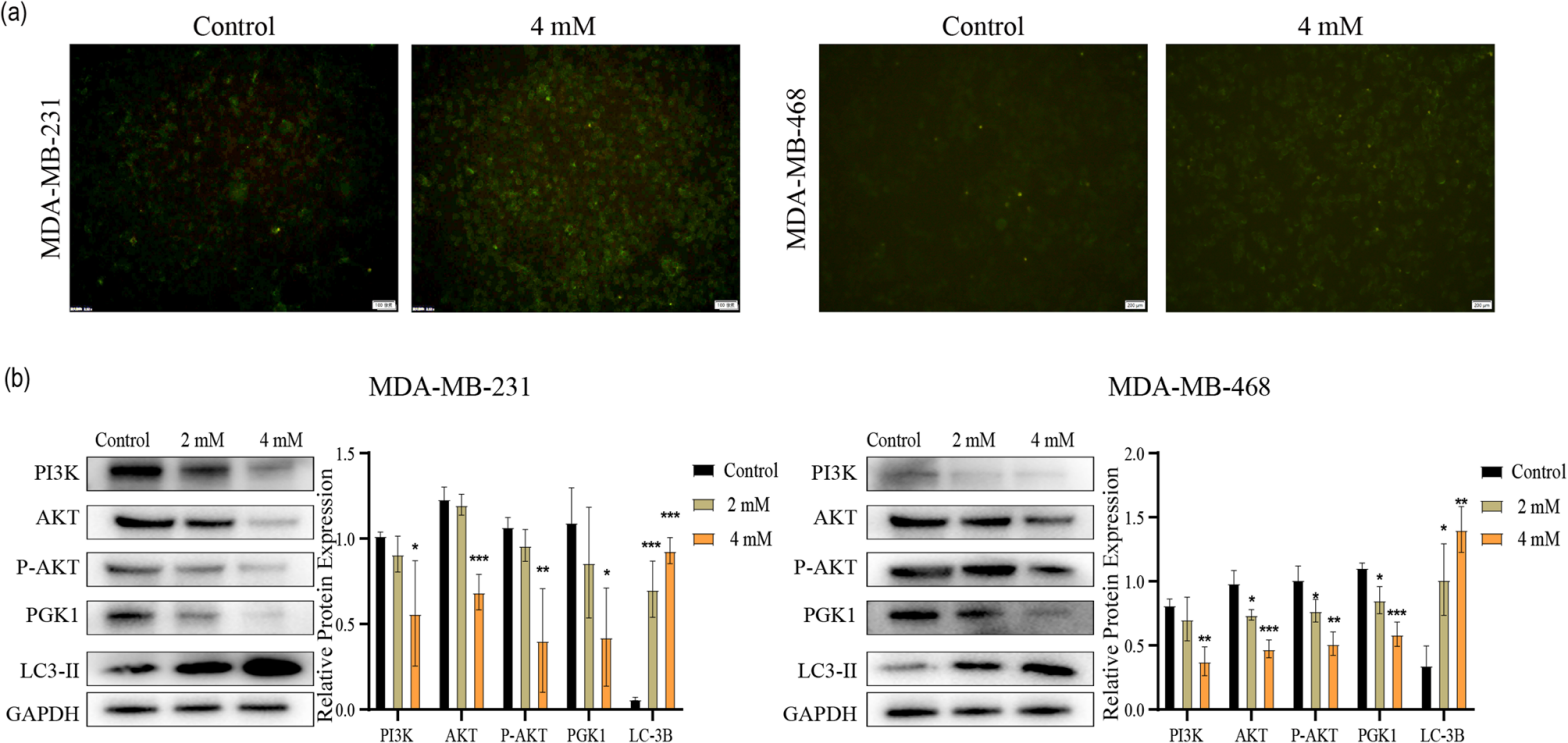
Effects of matrine (MAT) on autophagy and protein expression in TNBC (**a**) MDC staining was used to detect autophagy in the cells. (**b**) Western blot analysis was performed to determine the expression levels of PI3K, AKT, P-AKT, PGK1, and LC3B-II in TNBC cells. The original imaging representing immunoblots ware shown in Supplementary Fig. S2.

To verify if MAT can induce autophagy, we performed MDC staining experiment. MDC is a fluorescent dye that can be taken up by cells and display autophagic vacuoles, with clear punctate structures visible in the cytoplasm or perinuclear regions. Therefore, the level of intracellular autophagy can be determined according to the changes in the intracellular particles under a fluorescence microscope. Compared with the control group, the MAT (4 mM)-treated group had a higher density and more labeled autophagic granules (Fig. 6a). This finding indicates that MAT significantly induced autophagy.

### MAT inhibits predictive protein expression

After treating TNBC cells with MAT (0, 2 and 4 mM) for 24 h, the expressions levels of PI3K, AKT, P-AKT, and PGK1 significantly decreased, whereas those of LC3-II significantly increased (Fig. 6b). LC3-II is involved in autophagy development, and it can be used to monitor autophagy activity without any exogenous markers. The assessment of LC3-II expression is one of the main autophagy detection methods^33^. The increased expression of LC3-II showed that autophagy significantly increased. This indicated that MAT can promote autophagy. These studies showed that MAT inhibits the PI3K/AKT pathway and promotes autophagy in TNBC cells.

## Discussion

TNBC is associated with a high recurrence rate and metastasis, compared with other types of breast cancer. Chemotherapy is the main treatment method for TNBC, but patients are prone to drug resistance, which reduces treatment efficacy^34^. MAT has an inhibitory effect on a variety of cancers, including lung, gastric, colon, and liver cancers, as well as acute leukemia^35–39^. MAT can enhance the inhibitory effects of afatinib on H1975 cells^40^. The use of chemotherapy drugs alone and in combination with other strategies for breast cancer treatment have been compared; for instance, patients treated with the combination of compound Kushen injection and chemotherapy drugs showed reduced adverse drug reactions than patients treated with chemotherapy alone^41^.

Apoptosis is programmed cell death regulated by genes and mainly manifests as cell membrane contraction, nuclear rupture, and chromatin marginalization. After the TNBC cells were treated with MAT, the cells began to undergo atrophy and deformation, and vacuoles appeared in the cells. Flow cytometry showed that with the increase in MAT concentration, compare with that of the control group, the percentage of apoptotic TNBC cells was significantly higher, and the mechanism of apoptosis is the regulation of cleaved caspase-3 and BCL-2. Cleaved caspase-3 is the activated form of Caspase-3, is one of the proteins involved in cell apoptosis. When apoptosis begins, caspase-3 catalyzes poly ADP ribose polymerase and acetyl-devd-7-amino-4-methylcoumarin and is activated to become cleaved caspase-3. This process inhibits DNA repair and promotes apoptosis. Bcl-2, which plays an important function in the process of resisting tumor apoptosis, is an anti-apoptotic protein with key functions. Bcl-2 can set the threshold for cell apoptosis through the mitochondrial membrane and block the cascade of caspases, thereby inhibiting programmed cell death^42,43^. This indicates that MAT can effectively induces TNBC cells apoptosis.

The slowdown in tumor cell proliferation may be related to the arrested cell cycle. The streaming cell technology verifies that MAT affects cell cycle by arresting cells at the G0/G1 stage. Cell cycle disorders are the basis of the impaired proliferation of cells, and they also promote changes in the instability of genetic information^44^, thereby promoting carcinogenicity. Therefore, targeted regulation of the cell cycle may be an effective cancer treatment strategy^45^. MAT arrests the process of cell replication, which may interfere with the disorder of cell cycle of cancer cells and promote its apoptosis. New studies have found that the combined application of cycle inhibitors and other chemotherapy drugs can improve the efficacy of chemotherapy^46^.

Cell autophagy is the process of self-degradation and recreation of intracellular aging organelles and toxic components that plays a regulatory function in maintaining the steady state of cells. Compared with the control group, the MAT (4 mM)-treated group formed denser and brighter particles after MDC staining. Western blotting showed that the expression of the autophagosome structural protein LC3-II increased in the MAT group, which proved that MAT could induce TNBC cells to produce more autophagosomes, and this induction was concentration-dependent. Autophagy is regulated by mammalian target of rapamycin (mTOR), which is a downstream signal of the PI3K/AKT pathway.

The cooperation of network pharmacology, disease molecular big data, and computer auxiliary simulation can reveal the potential of natural products^47^. We selected the DEGs of normal breast and TNBC through bioinformatics, and PGK1 and PI3KCA ware identified after carrying out a prognostic value analysis. PGK1 can transfer the phosphate group from 1,3-diphosphoglycerate to 5′-adenosine diphosphate and generate ATP^48^. PGK1 is an essential enzyme for glycolysis and mitochondrial metabolic pathways. PGK1 is dynamically regulated by O-GlcNAcylation, and glycosylation on T255 activates its enzyme activity, inhibiting pyruvate metabolism^49^. PGK1, as a glycolytic enzyme and protein kinase, plays an important role in the regulation of cellular metabolism and autophagy in maintaining cellular homeostasis^50^, and may also be intervened by the PI3K/AKT pathway^51,52^. Its mRNA and protein expression levels are upregulated in various clinicopathological types of breast cancer^53^. High expression of PGK1 in cells significantly correlated with poor prognosis of cancer^54^. Some studies have confirmed that long noncoding RNAs (lncRNA) can promote tumor metastasis via PGK1-activated AKT/mTOR pathway^51,52^.

Both NG52 and MAT can bind near ASN35. The binding affinity of NG52, as a kind of positive control drug, is better than that of MAT (− 7.565 kcal/mol < − 6.120 kcal/mol). Although the binding ability of MAT and PGK1 is not as good as that of NG52 and PGK1, MAT can not only interfere with PGK1, but also combine with PIK3CA. In the realm of current clinical treatments, drugs with a single acting target have begun to exhibit limitations, notably the burgeoning resistance among patients. This growing resistance results in diminishing benefits for patients utilizing these types of medications^55^. By inhibiting PI3K/AKT pathway, MAT plays its anti-tumor function and has a broader biological activity. If it is a PGK1 resistant patient, it may have unexpected effects in clinical treatment. In addition, NG52 was discovered through network pharmacology and its inhibitory effect was verified in vitro^32,56^. NG52 or related products are rarely used in human research in PubMed. MAT is derived from the natural plant *Sophora flavescens*, which is widely used in Traditional medicine in China^57^. Studying MAT not only clarifies the mechanism of *Sophora flavescens* in treating cancer, but also provides a basis for the development of new drugs, hoping to bring more effective treatment effects to patients while reducing their burden. After MAT intervention in TNBC cells, western blotting confirmed the downregulation of PGK1. When the expression of PGK1 is inhibited, the intracellular glycolysis process is inhibited. Based on the above results and analysis, we believe that MAT has the potential to become a new inhibitor of PGK1, but its mechanism needs further research.

Currently, in the era of the search for precise treatment strategy for tumors, the clinical trend is to select appropriate treatment plans through personalized diagnosis of patients, prevent the spread of cancer by inhibiting specific genes or signaling pathways, and improve their quality of life. It is of great significance to study the abnormal expression of genes associated with cancer-related pathways to inhibit the occurrence and progression of tumors.

One of the most frequent abnormal changes associated with cancer occur in the PI3K/AKT signaling pathway and is an important node in many cancers. The inhibition of the PI3K/AKT pathway can effectively suppress various cancers, including ovarian, non-small cell lung, and breast cancers^58–60^. PI3K/AKT pathway inhibitors can enhance the therapeutic effect of chemotherapy drugs^61^, and some inhibitors of this pathway have also been verified in clinical trials^62^. The PI3K/AKT pathway regulates a wide range of cellular processes, including cell survival, proliferation, growth, metabolism, angiogenesis, and metastasis^63^. It exhibits integral roles in cell metabolism, autophagy, apoptosis, and cell cycle regulation. The PI3K/AKT pathway has broad prospects for the treatment of TNBC. PIK3CA is one of the two most common oncogenes in human tumors, the alpha catalytic subunit of PI3K, which plays an important role in regulating the PI3K pathway^27^. The activation mutation of PIK3CA can upregulate the PI3K/AKT signaling axis to promote tumor development, and has recently been identified as a new mechanism for inducing carcinogenic PI3K signaling^64^. Data shows that PIK3CA mutation plays a promoting role in many patients with breast cancer^65^.

**Figure 7:**
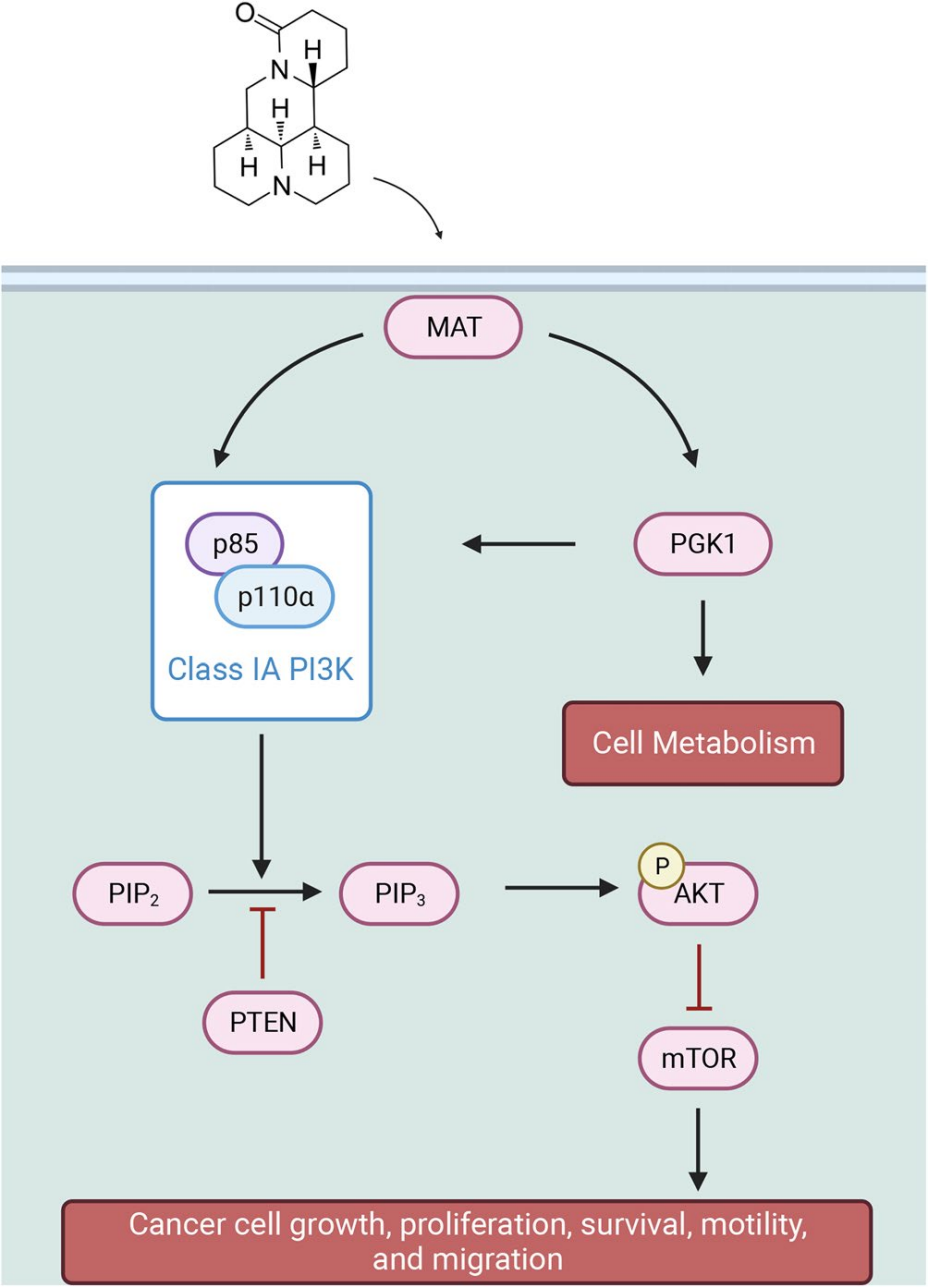
MAT and PI3K/ AKT signaling pathway.

Some proteomics studies have shown that abnormally expressed phosphorylated AKT plays a negative role in tumors^66,67^. This study also demonstrated that MAT inhibits the expression of P-AKT. After MAT intervention in PI3K signaling, it inhibited the expression of downstream AKT and reduced AKT phosphorylation levels. With increasing MAT concentration, the invasion and migration capabilities of TNBC cells significantly decreased. Therefore, our results suggest MAT activates autophagy and promotes tumor cell apoptosis by inhibiting the PI3K/AKT pathway, which provides pharmacological evidence for the use of matrine in many traditional Chinese medicine prescriptions for cancer treatment (Fig. 7).

Although *Sophora flavescens* is a commonly used medicine in traditional Chinese medicine and is widely used in the clinical anti-tumor process, its intervention mechanism is still not very clear. In future studies, we hope to explore the efficacy of MAT in tumor animal models by combining PI3K activators/inhibitors. In this study, we found that PGK1 may also be regulated by MAT, resulting in anti-tumor activity, which is rarely reported in MAT related studies. We expect to delve deeper into the mechanism of MAT inhibiting PGK1 mediated tumor cell metabolism in our next research.

## Conclusion

MAT inhibited the PI3K/AKT pathway, downregulated PGK1 expression. MAT also inhibited the growth of TNBC cells, caused cell death, activated apoptosis, arrested the TNBC cell cycle in the G0/G1 phase, and induced autophagy. This study demonstrated an efficient method to explore natural drug interventions in TNBC through network pharmacology and in vitro experiments.

## Supplementary Information

Supplementary Information.

## Data Availability

The datasets (GSE41970, GSE45827, and GSE65194) analyzed during the current study are available in the GEO database (www.ncbi.nlm.nih.gov/geo/).
