# Supplementary material for: MAT as a promising therapeutic strategy against triple-negative breast cancer via inhibiting PI3K/AKT pathway
Source: Sci Rep. 2023 Jul 31;13:12351. doi: 10.1038/s41598-023-39655-9 (PMC10390516; doi:10.1038/s41598-023-39655-9)
Supplement: Supplementary file 1 — Supplementary Information. [file 41598_2023_39655_MOESM1_ESM.pdf]

Supplementary Information

MAT as a promising therapeutic strategy against triple-negative breast cancer via inhibiting PI3K/Akt pathway

S1:

C-cap-3 MDA-MB-231 MDA-MB-468

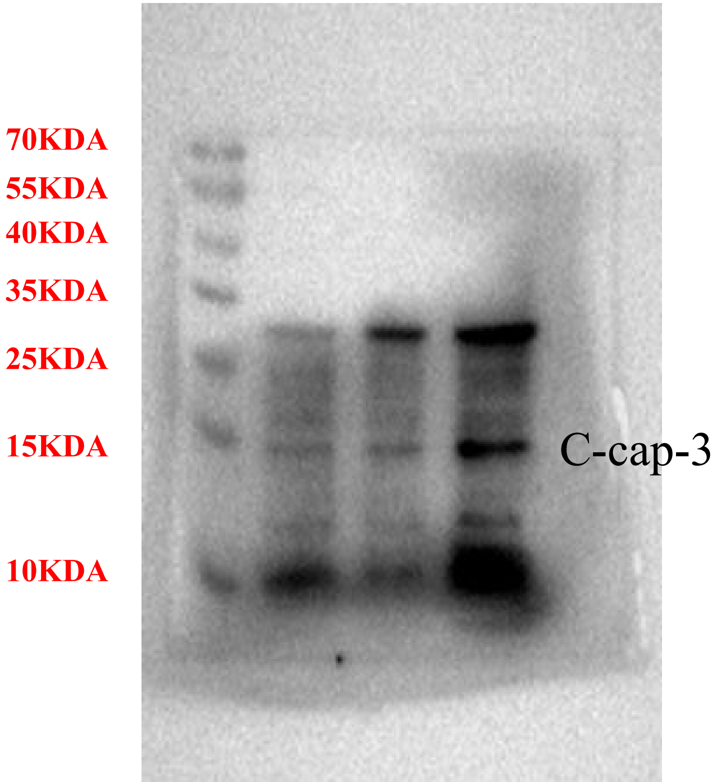

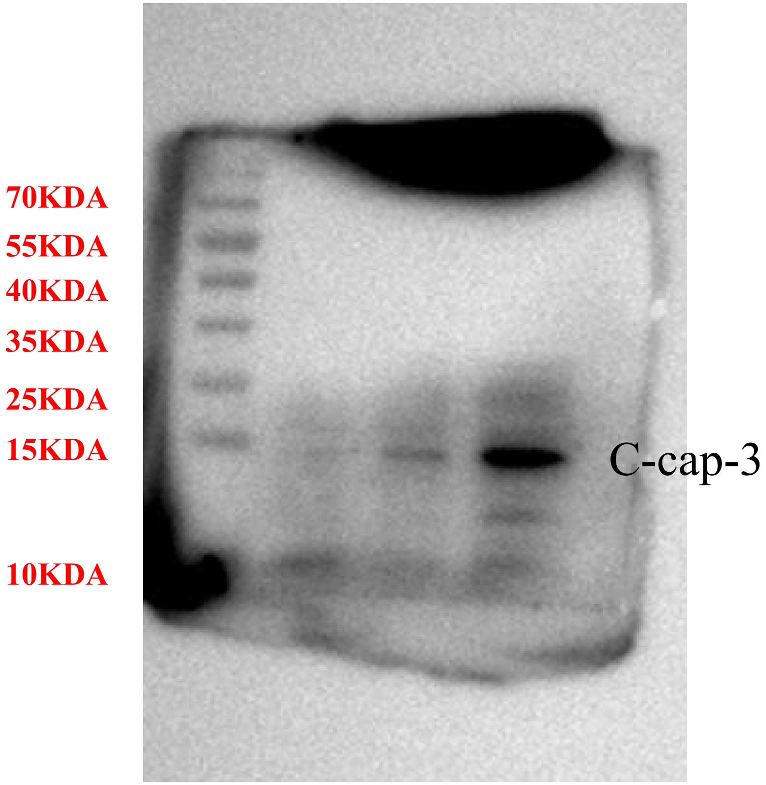

BCL-2

MDA-MB-231

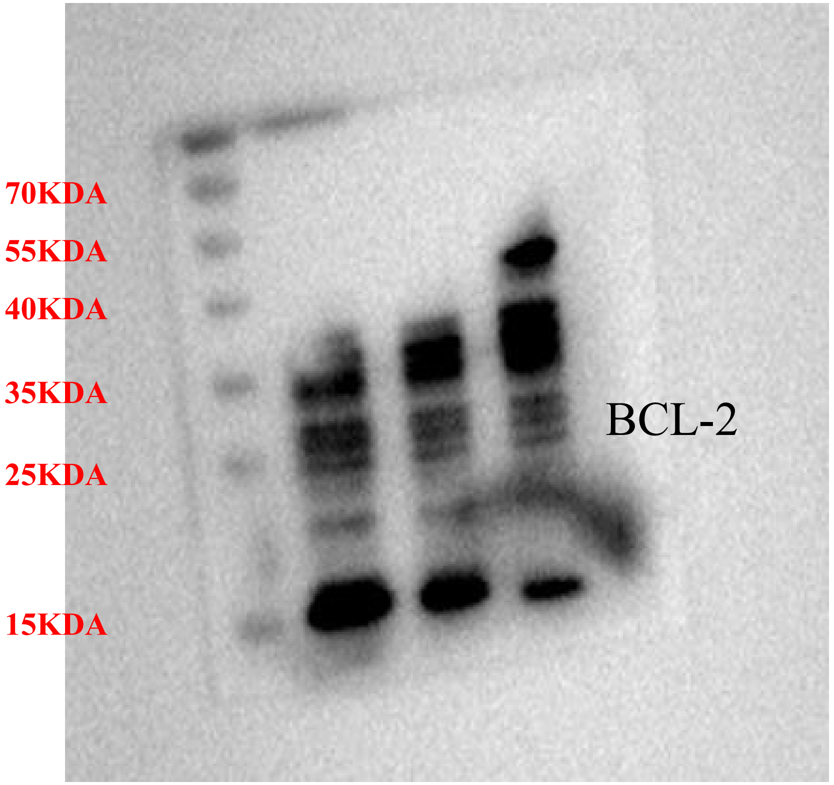

MDA-MB-468

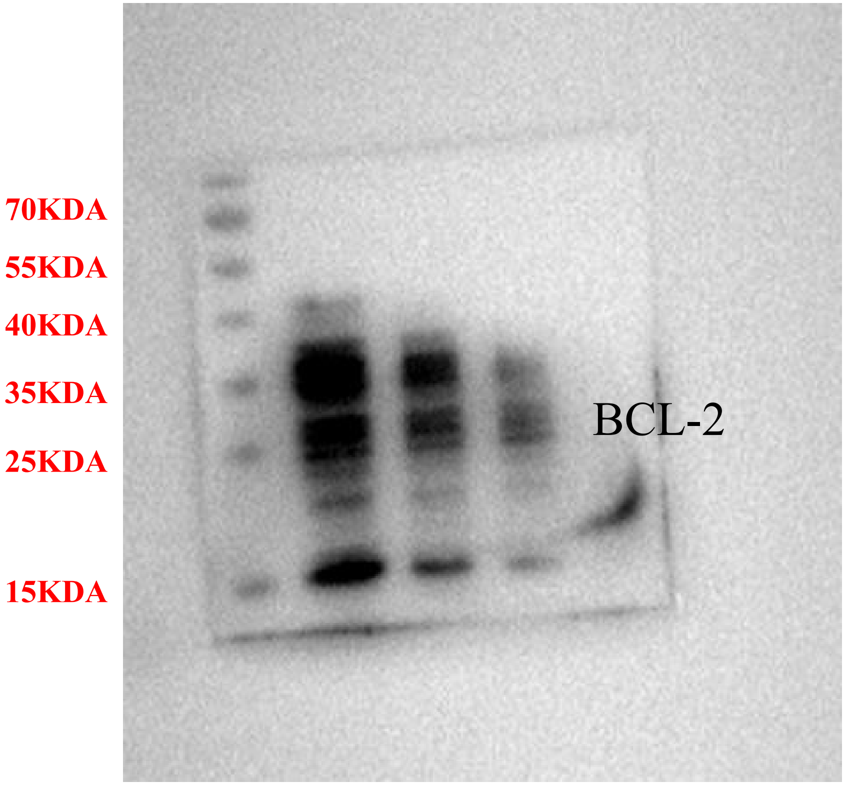

GAPDH MDA-MB-231 MDA-MB-468

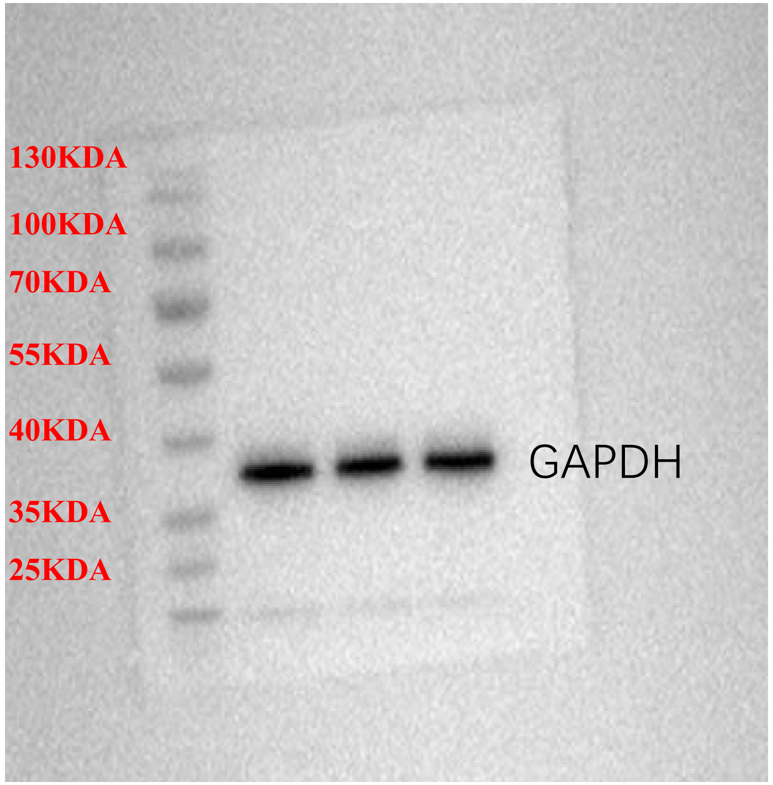

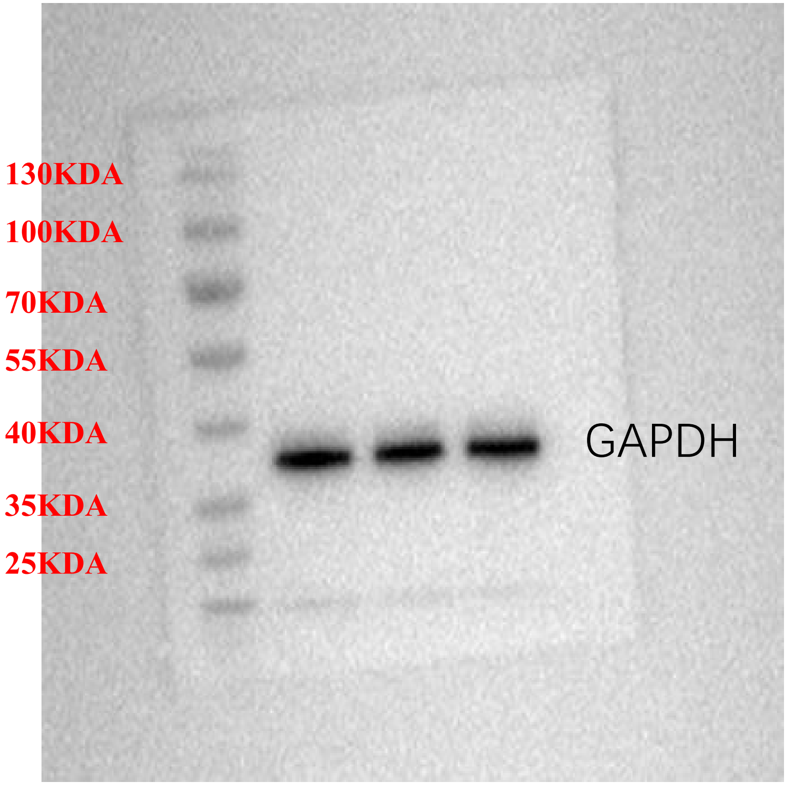

Supplementary FigureS1.The original imaging representing immunoblots of Fig. 3.

S2:

PI3K

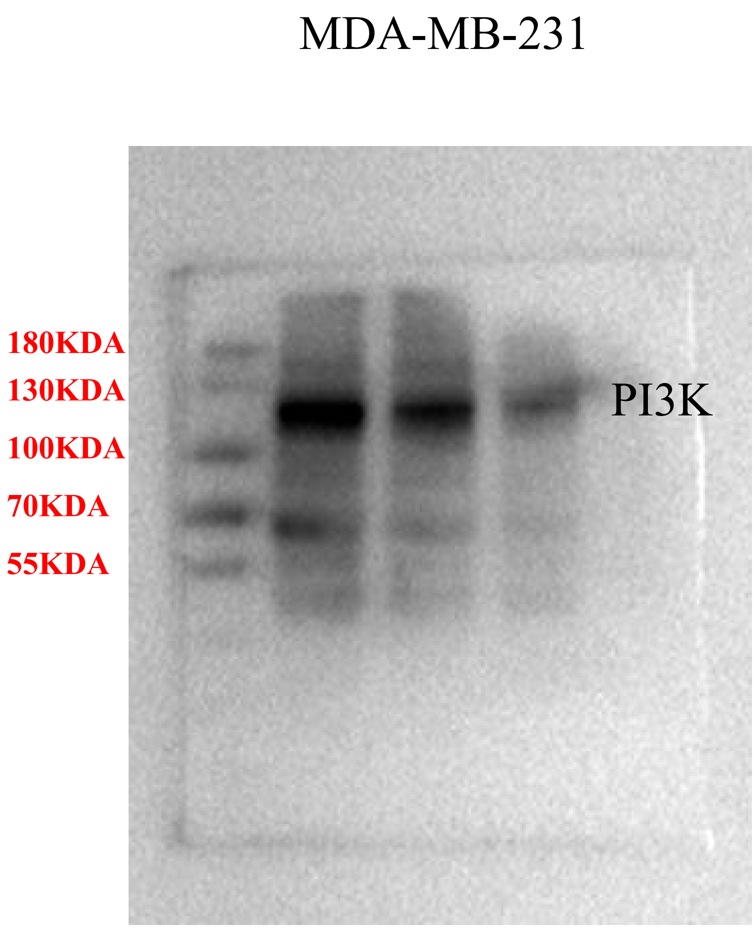
MDA-MB-231
180KDA
130KDA
100KDA
70KDA
55KDA
PI3K

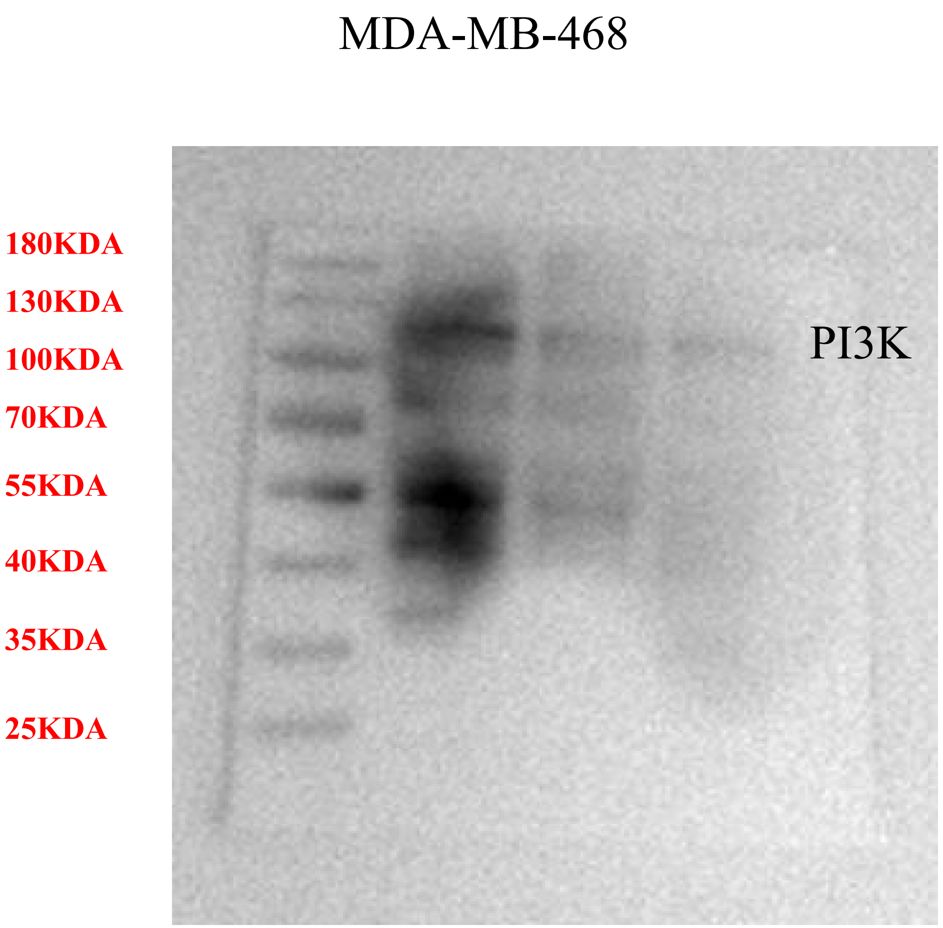
MDA-MB-468
180KDA
130KDA
100KDA
70KDA
55KDA
40KDA
35KDA
25KDA
PI3K

P-AKT

MDA-MB-231

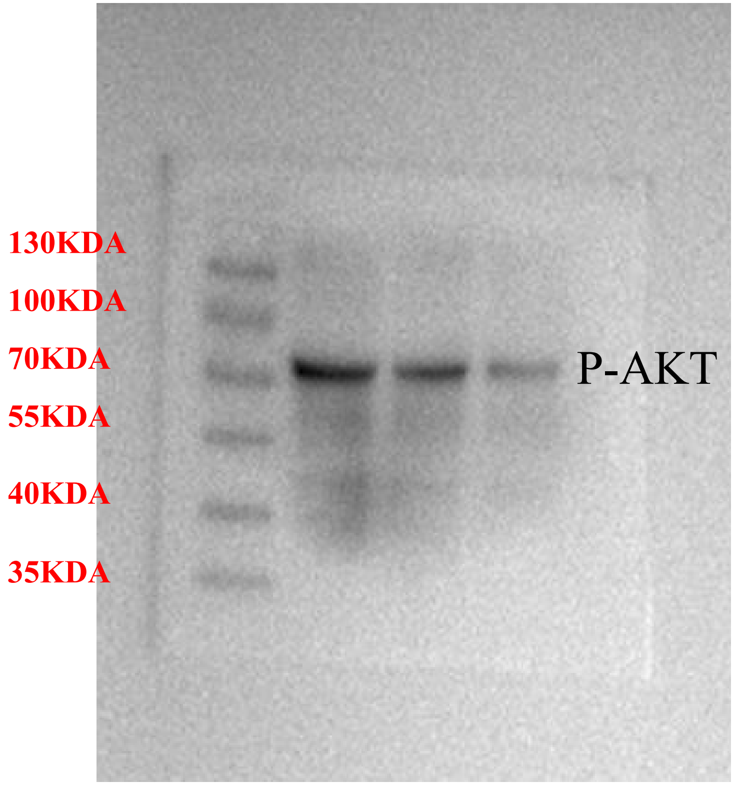

MDA-MB-468

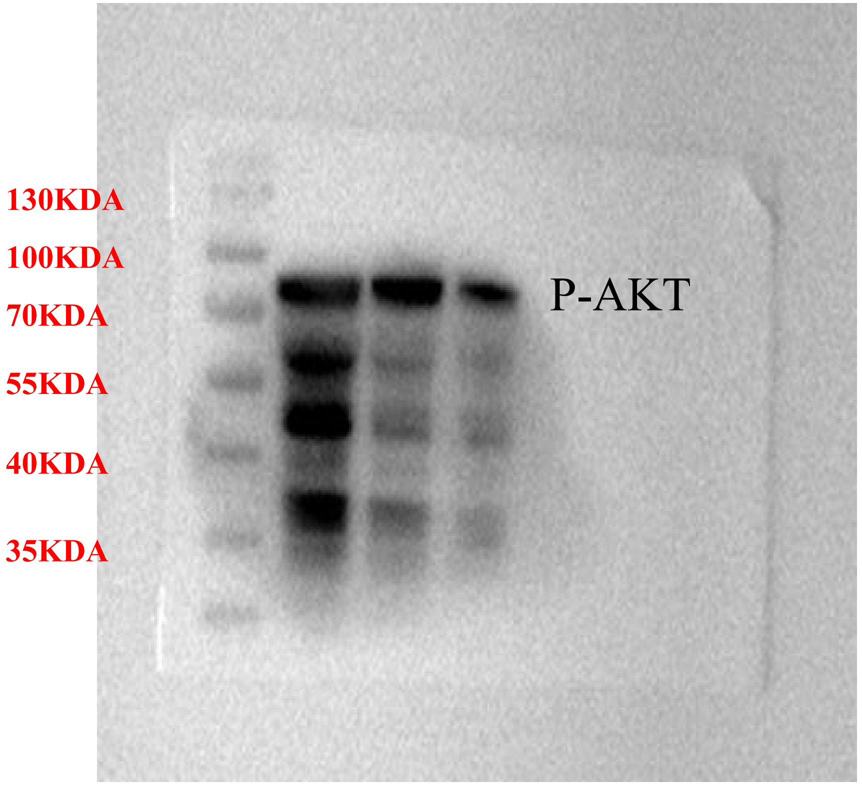

AKT

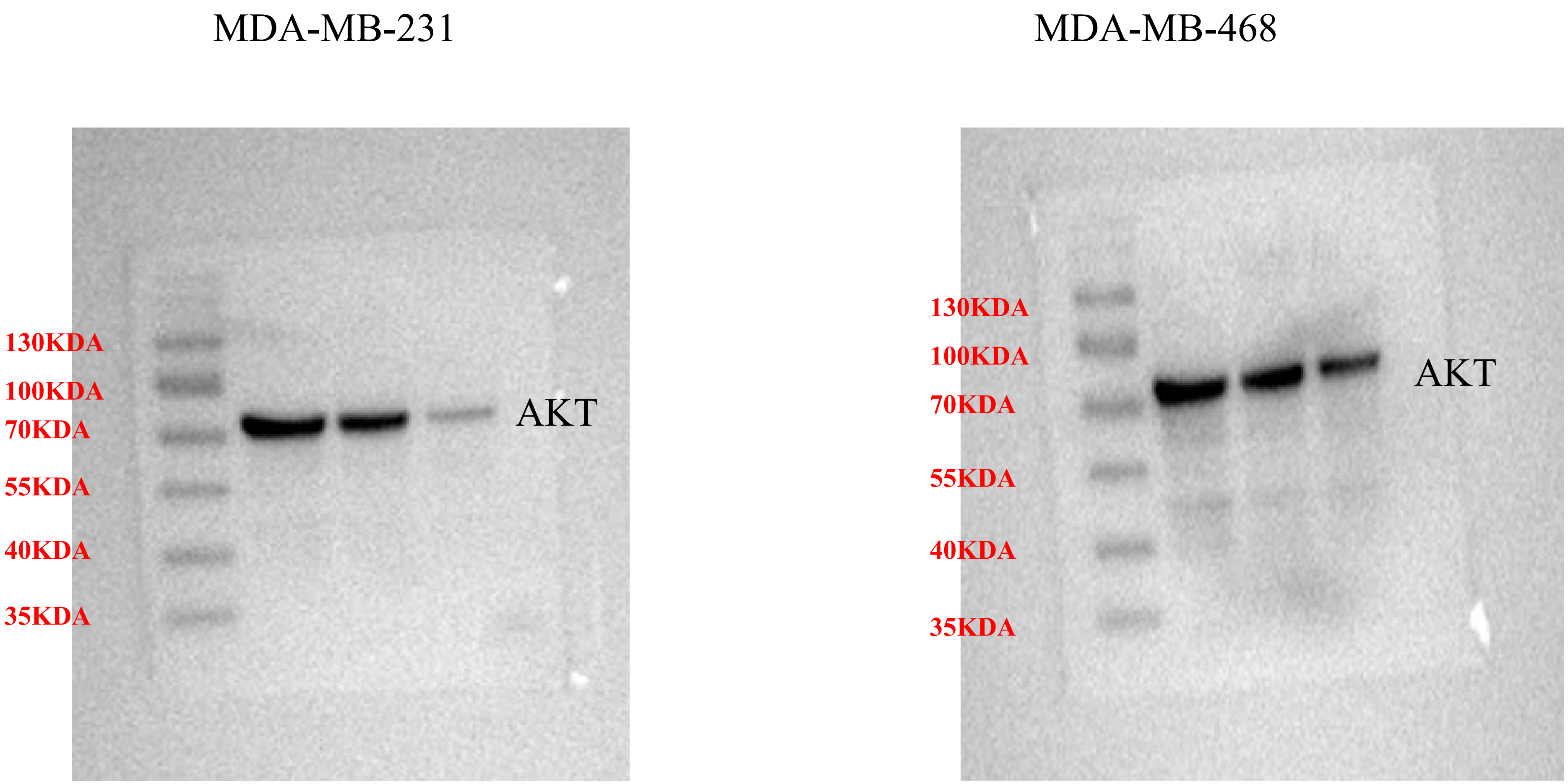

MDA-MB-231
130KDA
100KDA
70KDA
55KDA
40KDA
35KDA
AKT
MDA-MB-468
130KDA
100KDA
70KDA
55KDA
40KDA
35KDA
AKT

PGK1

MDA-MB-231

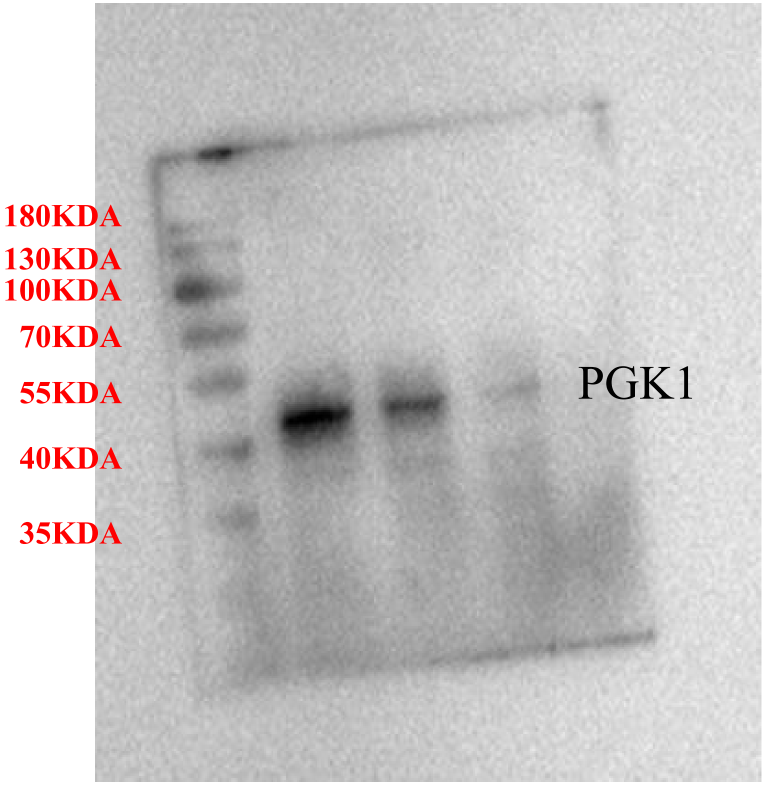

MDA-MB-468

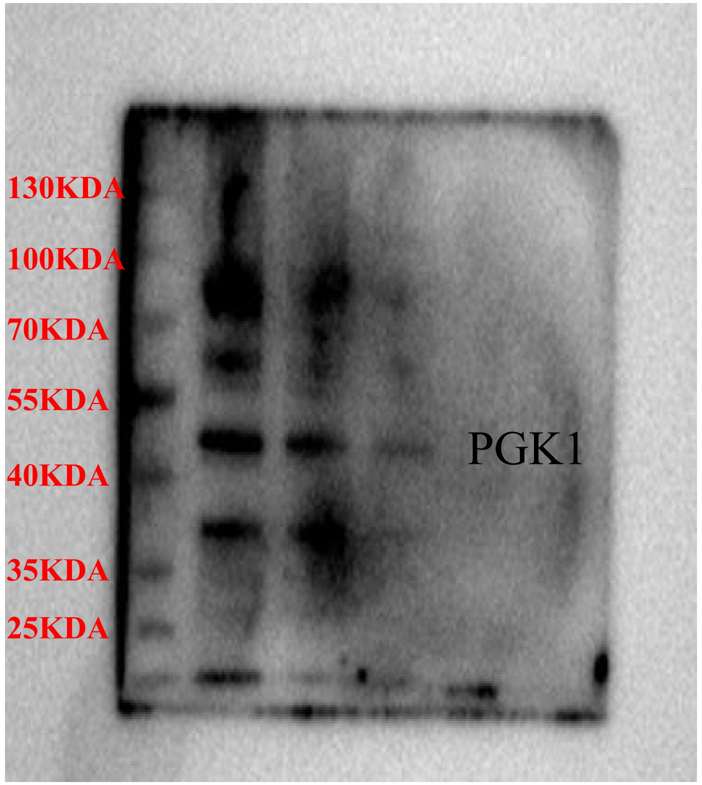

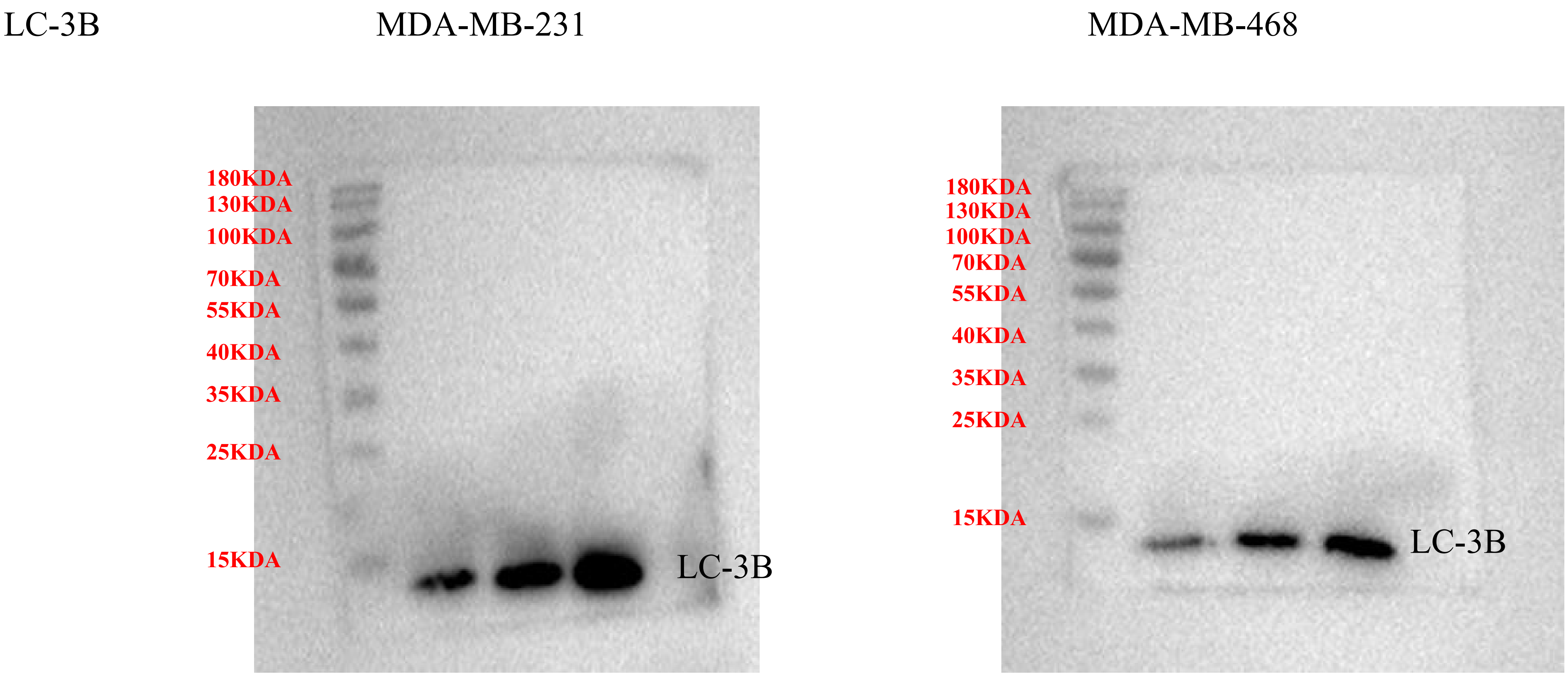

Supplementary FigureS2.The original imaging representing immunoblots of Fig. 6.

GAPDH

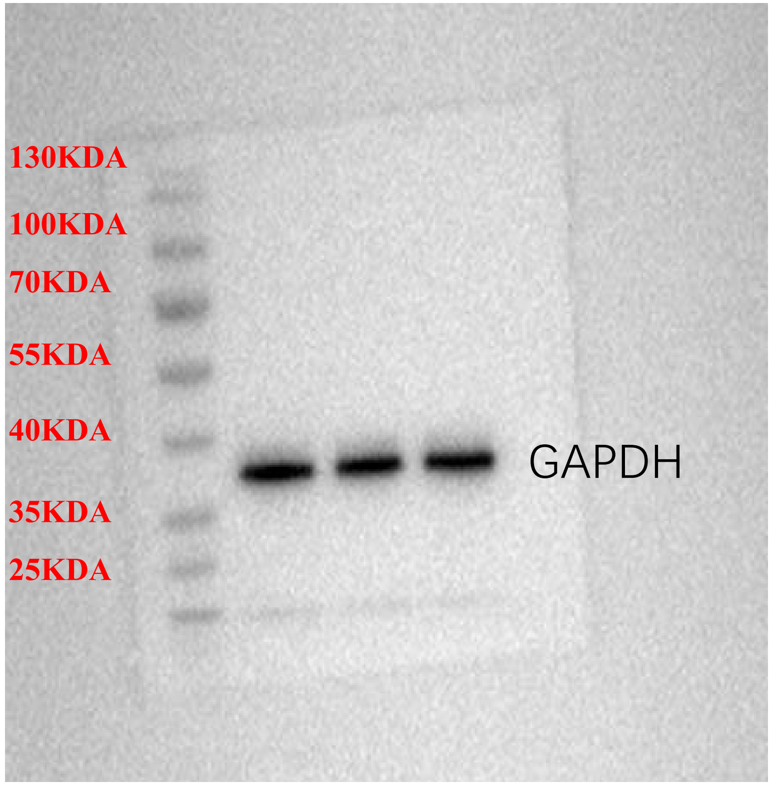

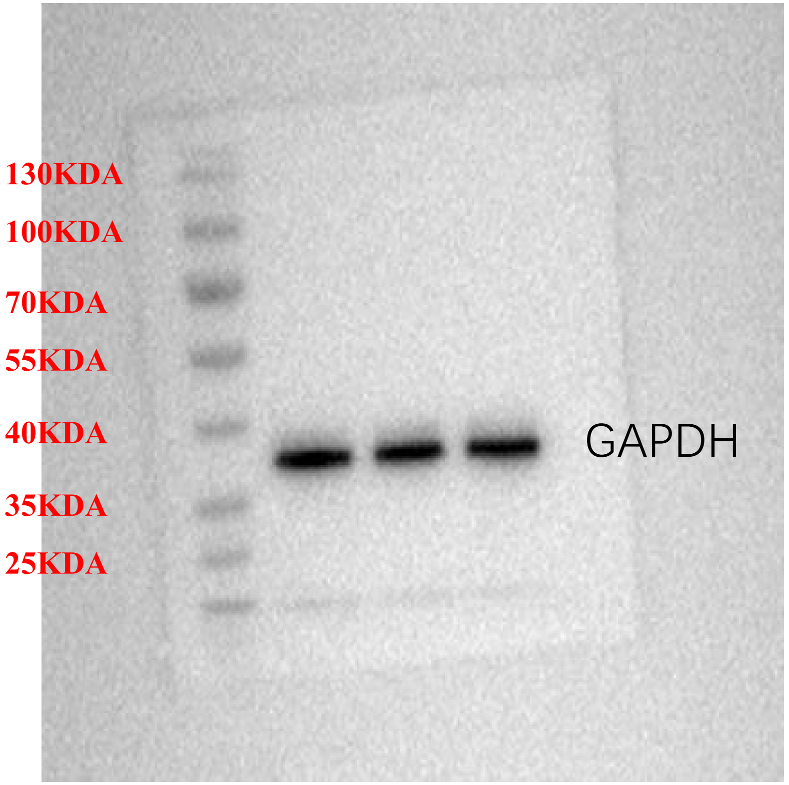

Supplementary FigureS2.The original imaging representing immunoblots of Fig. 6.
